# Changes in climate attributes and harvest area structures jointly determined spatial-temporal variations in water footprint of maize in the Beijing-Tianjin-Hebei region

**DOI:** 10.1016/j.heliyon.2024.e32565

**Published:** 2024-06-06

**Authors:** Heju Huai, Qian Zhang, Min Liu, Xiumei Tang

**Affiliations:** a Information Technology Research Center, Beijing Academy of Agriculture and Forestry Sciences, Beijing, 100097, China; b National Engineering Research Center for Information Technology in Agriculture, Beijing, 100097, China

**Keywords:** Beijing-Tianjin-Hebei, Irrigation, Maize production, Rainfall, Water use efficiency

## Abstract

Irrigation contributes significantly to boosting crop yield and ensuring food security. However, in the Beijing-Tianjin-Hebei (BTH) region, unsustainable irrigation practices have led to serious outcomes on freshwater resources. Balancing irrigation with crop productivity in this region, currently facing complex challenge, requires a comprehensive understanding of its spatial pattern and thus to seeking for potential optimization of current crop structures. In this study, we employed the concept of water footprint (WFP) to assess the spatial-temporal patterns of water footprint for maize in BTH region at the county level for the years 2005, 2010, 2015, and 2020, untangled the relative impacts on WFP from climate attributes and harvest area structures. Our results showed significant regional heterogeneities in both blue water requirement and green water requirement, ranging from 64.6 mm to 290.7 mm. Yearly anomalies of climate attributes and maize harvest jointly influenced water footprints, with the highest value of 1.06 × 10^11^ m^3^ occurring in the year 2015. The green water footprints, linked to precipitation, dominated the total water footprint compared to the blue water footprint associated with irrigation. Additionally, we observed an increasing influence of maize harvest area on the temporal changes in water footprints, with these changes becoming more concentrated in the east-central region over time. Our findings underscore the respective contributions of annual climate attribute changes and harvest area variations at the county level, highlighting regions where urgent interventions are required to enhance the sustainability of water usage for agriculture.

## 1 Introduction

Water scarcity is a pressing global issue that has profound implications for economies and the quality of human life in numerous countries. The challenges posed by water scarcity have become even more pronounced due to factors such as a rapidly growing population, increasing demands for food and animal feed, and evolving, water-intensive consumption patterns that have emerged during the Anthropocene era. Currently, over 70 % of the world's freshwater withdrawals from rivers, lakes and aquifers are allocated for agricultural irrigation to sustain and enhance agricultural productivity [[1], [2], [3]]. Agriculture is presently confronted with a dual challenge, the need to mitigate water shortages while simultaneously enhancing agricultural productivity. These challenges are further compounded by the effects of climate change and shifts in dietary habits driven by socioeconomic development [4]. Consequently, it is of paramount importance to accurately ascertain crop water requirements and their relationship to crop production. This precision is vital for policymakers as they formulate strategies to effectively address these multifaceted challenges.

Water footprints (WFPs) is a strong tool for quantifying the amount of water resources needed for all the goods and services used over a specific time period [5]. WFPs can be divided into three categories. Green water footprint (WFP_green_) reflects the portion of rain water that does not become surface runoff or contribute to other forms of water loss. Blue water footprint (WFP_blue_) represents the consumption of surface water (e.g., rivers and lakes) and groundwater. Grey water footprint indicates the level of water pollution as the volume of water required to assimilate the load of pollutants given natural background concentrations and existing ambient water quality standards [6]. The water footprints (WFPs) are commonly expressed as either the water volume per year of a delineated area or region (m^3^/yr) or the water volume required to produce a unit product (m^3^/t). Subsequently, a number of studies focusing on the WFPs of different crops. Such as, cereal crops, wheat and rice at global scale [7,8], wheat, maize, and rice at national scale [9,10] and region scale [11,12], cash crops, soybean, onion, and carrot [[13], [14], [15], [16]]. As an evaluation index of water resource exploitation, WFPs has been linked to regional water resources and water environment sustainability in research practice [17,18]. Studies have shown that reducing the WFPs in agriculture is an effective strategy for addressing local water shortages and promoting ecological sustainability [11,19].

In China, the rapid growth of the economy and the escalating demand from both agricultural and industrial production have exerted significant pressure on the water environment. The issue of water scarcity is particularly pronounced in Northern China, notably in the Beijing-Tianjin-Hebei (BTH) region [20], where the available water resource has declined significantly from 28 to 29 billion m^3^ in the late 19th century to 14 to 15 billion m^3^ in the early 21st century [21], and the per capita water resources in this region are less than 300 m^3^ per year, far below the internationally recognized water scarcity threshold of 1700 m^3^ per year [22]. Simultaneously, the BTH region stands out as an essential agricultural production area in China, characterized by its rich and fertile soil and ample solar radiation. Nevertheless, the region heavily relies on groundwater as the primary source for crop irrigation, resulting in excessive groundwater exploitation and the depletion of groundwater reserves. Consequently, the imperative to enhance water use efficiency and rationalize water resource allocation within the agricultural sector has become a pressing concern for the BTH region.

Maize is one of the primary crops in northern China, known for its consistently high yields. In recent years, the maize cultivation area has surpassed that of winter wheat, making it the largest crop in the BTH region. Furthermore, the growing season for maize matches well with the rainy season in the BTH region [23], which reduce the irrigation demand for maize production. The combination of high and consistent yields and low water costs has made maize not only crucial for ensuring food production in the BTH region but also for sustaining water resources. Furthermore, climate change is already a global issue that has dramatically affected maize production [24] by influencing the cycling of water resources [25]. The regional differences in the response of crop production to climate change will become stronger [26]. However, there is still a lack of information on the impact of climate change on the water footprint of maize production, as well as the joint effect of climate change and harvest area structures on the dynamics of water footprints in the BTH region [27,28]. Understanding the response of the spatial-temporal dynamics of water footprint for maize production to climate change would help in policy and decision-making. This could aid in formulating efficient strategies for conserving water resources and building sustainable agricultural systems under limited water resource conditions and climate change. Therefore, in this study, we investigated the crop water use for maize production in the BTH region through the water footprint indicator across 163 counties in the BTH region. The objectives are as follows: 1) to assess the crop water requirements for maize production in the BTH region, 2) to quantify the temporal and spatial variations in the green and blue water footprint of maize production for the years 2005, 2010, 2015 and 2020, 3) to explore the effect of changing climate and harvest area structures on the water footprint of maize production in the BTH region, and 4) to explore strategies for conserving water resources to support sustainable maize production in the BTH region.

## 2 Material and methods

### 2.1 Study region

**Fig. 1:**
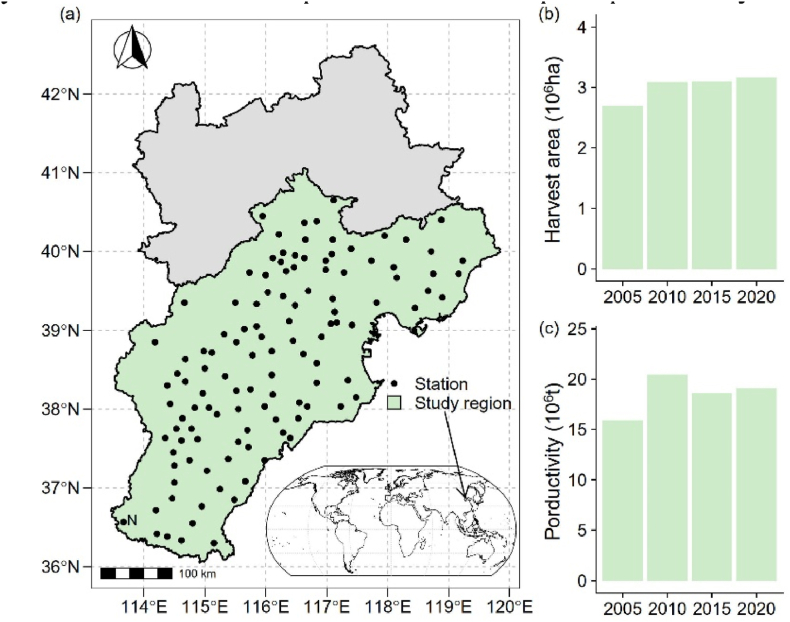
Study region (a), maize harvest area (b), and maize productivity (c) over the years 2005, 2010, 2015 and 2020.

The Beijing-Tianjin-Hebei region (BTH, 36°03′– 40°29′ N, 114°13' – 119°27′ E) is located on the coast of the Bohai Sea (Fig. 1a) with a total area of 21.67 million ha. Characterized by a temperate semi-arid monsoon climate, this region experiences an annual average temperature of 11.3 °C, coupled with an annual precipitation of 549 mm. The soil at the study area was mainly consist of Fluvisol based on the soil map from the Harmonized World Soil Database (v 1.2). Notably, maize cultivations in BTH yield more than half of the total grain production (Fig. 1 b, c), and serves as a pivotal maize production region in China. Generally, maize planted in BTH can be classified into spring maize and summer maize according to its planting date. We only focused on summer maize owing to its dominant status for maize production. Summer maize cultivation typically occurs in early June and harvests in late September, synchronized with the peak of seasonal precipitation cycles.

### 2.2 Data sources

We collected maize yield, harvest area, and productivity data for the years 2005, 2010 2015 and 2020 at the county scale from the Agricultural Statistical Yearbook, which is published yearly from sample surveys conducted in specific regions (https://data.stats.gov.cn/). These data were acquired from the Agricultural Information Institute of the Chinese Academy of Agricultural Sciences. Moreover, we collected maize phenological dataset, encompassing important phenological events, including the date of seeding, three leaves, seven leaves, jointing, booting, tasselling, flowering, silking, milk-ripening, and maturity, spanning the period from 1993 to 2013 across agro-meteorological stations. These agro-meteorological stations are maintained by the National Meteorological Information Centre (http://data.cma.cn/). Furthermore, we collected daily climate variables, including minimum temperature, maximum temperature, average temperature, precipitation, sunshine hours, wind speed, and relative humidity from 117 climate stations under the maintenance of the National Meteorological Information Centre (http://data.cma.cn/). To estimate potential evapotranspiration (*ET*_*0*_) across these climate stations, we employed the Penman-Monteith equation recommended by Allen et al. (1998) [29] according to the available weather data and the time step computation in Eq. (1).

(1) $${ET}_{0}=\frac{0.408\Delta \left(R_{n}-G\right)+\gamma \frac{900}{T+273}\mu_{2}\left(e_{s}-\;e_{a}\right)}{\Delta +\gamma \left(1+0.34\mu_{2}\right)}$$

where R_n_ is the net radiation at the crop surface (MJ m^−2^ day^−1^), G the soil heat flux density (MJ m^−2^ day^−1^), T the air temperature at 2 m height (°C), μ_2_ the wind speed at 2 m height (m s^−1^), *e*_*s*_ the vapour pressure of the air at saturation (kPa), *e*_*a*_ the actual vapour pressure (kPa), Δ the slope of the vapour pressure curve (kPa °C^−1^) and γ is the psychrometric constant (kPa °C^−1^). G may be ignored for daily time step computation.

### 2.3 Methodology

#### 2.3.1 Calculation of water requirement throughout maize growing season

The crop evapotranspiration process, influenced by the intricate dynamics of the soil–plant–atmosphere interface, encompasses varying evaporation and transpiration components under diverse environmental conditions. To quantify this, we employed the dual crop coefficient methodology as in Eq. (2).

(2) $${ET}_{c}=\left(k_{cb}\times k_{s}+k_{e}\right)\times {ET}_{0}$$

Where *ET*_*c*_ represents crop evapotranspiration for a specific crop, *K*_*cb*_ is the basal crop coefficient for a specific crop, which differed at different maize phenological stage, *K*_*s*_ is the water stress coefficient determined as a function of soil water content and soil water holding properties within the root zone, *K*_*e*_ is the evaporation coefficient, *ET*_*0*_ is the potential evapotranspiration (*ET*_*0*_) calculated using Eq. (1). We adopted the default *k*_*cb*_ parameters for maize during its growing season in Allen et al. (1998) [29]. Given the prevalent irrigation conditions for maize cultivation in this region, we assumed that maize is free of water stress, and thus set *k*_*s*_ as 1. *k*_*e*_ relies on dynamic assessments of water depletion within the surface soil layer [29].

Effective Precipitation (*Peff*) quantifies the amount of precipitation that can be retained in the soil after accounting for losses from runoff and ineffective evaporation [30]. We calculated *P*_*eff*_ during maize growing season based on USDA Soil Conservation Service method as cited by Smith (1992) [31] in Eq. (3).

(3) $$P_{eff}=\left\{ \begin{array}{c} P\times \frac{125-0.2\times 3\times P}{125}\;P\leq 83.3mm\\ \frac{125}{3}+0.1\times P\;P>83.3mm \end{array}\right.$$

Where *P* represents the total amount of precipitation in ten days (mm), 83.3 mm represented the threshold of precipitation whether effect of direct runoff volume dominated the total amount of precipitation.

The components of crop water requirement, including green water requirement and blue water requirement, can be determined by comparing their values during maize growing season using Eqs. (4), (5), (6).

(4) $$CWR={CWR}_{blue}+{CWR}_{green}$$

(5) $${CWR}_{blue}=\sum_{i=1}^{n}\max\left(0,{ET}_{c}-P_{eff}\right)$$

(6) $${CWR}_{green}=\sum_{i=1}^{n}\min\left({ET}_{c}{,P}_{eff}\right)$$

Where CWR, CWR_green_, and CWR_blue_ represent the total crop water requirement (mm), blue water requirement (mm), and green water requirement (mm), respectively. To extrapolate from the climate station scale to the county scale, we employed the thin plate spline interpolation method to upscale the irrigate water requirements (*IWRs*) [32].

#### 2.3.2 Calculation of water footprint during maize growing season

We applied the methodology developed by the Water Footprint Network to calculate the total water footprint [5,8], constituting by blue water footprint (WFP_blue_) and green water footprint (WFP_green_) as in Eqs. (7), (8), (9).

(7) $${WFP}_{s}={WFP}_{blue}+{WFP}_{green}$$

(8) $${WFP}_{blue}=10\times {CWR}_{blue}\times A$$

(9) $${WFP}_{green}=10\times {CWR}_{green}\times A$$

Where WFP_s_, WFP_blue,_ and WFP_green_ represent the total (green and blue) water footprint (m^3^), blue water footprint (m^3^) and green water footprint (m^3^), respectively; CWR_blue_ and CWR_green_ represent the blue and green water requirements, respectively; A represents the harvest area (ha) of maize within a specific county; The constant 10 was employed to convert water depth (mm) to water volume per unit area (m^3^ ha^−1^).

To assess the water footprint per unit of maize produced, we calculate WFP_y_ by dividing WFP by maize production as in Eq. (10).

(10) $${WFP}_{y}=\frac{WFP}{Y}$$

Where WFP_y_ is the water footprint per unit maize product (m^3^ t^−1^) and Y is the maize productivity (t) within a specific county.

#### 2.3.3 Spatial interconnections and drivers for WFPs dynamics

To assess the spatial connections among different facets of the WFPs, we calculated Moran's I, introduced by Moran (1950) [33], at local scales. Moran's I reflects the extent of spatial dependence among variables within a given geographic area, making it a valuable tool for assessing the interconnections within the water footprint for crops [34,35]. We robustly tested the global Moran's I statistic by using Monte-Carlo simulation. To further reflect the spatial associations of local indicators, we employed the local autocorrelation analysis (LISA), which combines local Moran's I with significance testing at *P* < 0.05 level across all counties. This analysis categorizes clusters into high–high clusters, low–low clusters, low–high outliers, and high–low outliers, as originally defined by Anselin (1995) [36]. In details, high-high clusters denote areas with high WFPs values surrounded by similar high-value regions, indicating concentrated water consumption region, while low-low clusters signify areas with consistently low WFPs values, suggesting low water consumption. And high-low outliers reveal areas with low WFPs values amidst regions of high water usage, while low-high outliers highlight locations with high WFP values surrounded by regions with lower water consumption.

Furthermore, we designed two scenarios to enhance our understanding of the impact of changes in maize cultivation area and crop water requirement due to climate change on the dynamics of water footprints. These scenarios involved assuming a fixed area across counties at the 2005 level (referred to as the “2005 area level”) and maintaining constant crop water requirements corresponding to the conditions at year 2005 (referred to as the 2005 WFP level). The comparison of the “2005 area level”, “2005 WFP level”, and the “baseline” scenarios allowed for a comparative analysis of WFPs dynamics, isolating the impact of changes in land use (harvest area) and water demand over time within the study region.

## 3 Results

### 3.1 Maize water requirement in BTH region

**Fig. 2:**
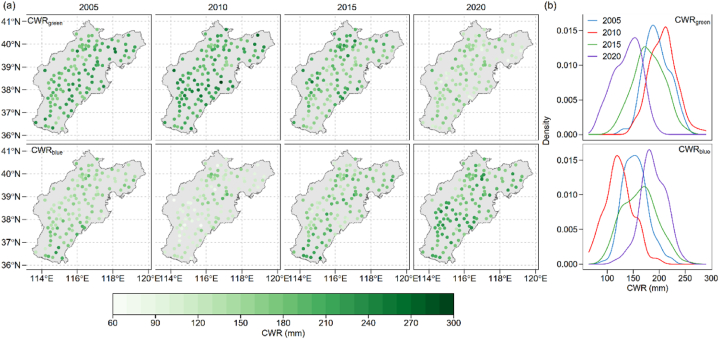
Crop water requirement and density of maize production in the BTH region (a) over year 2005, 2010, and 2015. The upper two panels indicate the green water requirement, and the lower two panels indicate the blue water requirement.

Our calculated green and blue crop water requirements showed that for maize production in the BTH region, among most of the observed climatic stations, green water requirement is higher than the blue water requirement (Fig. 2). The CWR_green_ ranges from 130.4 to 250.0 mm, 150.0–290.7 mm, 109.3–255.8 mm and 79.4–194.6 mm at year 2005, 2010, 2015 and 2020 respectively (Fig. 2a). Upon examining interannual variations in CWR_green_, we noted that during the maize growing season in 2020, the CWR_green_ was generally lower compared to the levels observed in the other study years, despite its considerable variability (Fig. 2b). The CWR_blue_ ranges from 93.4 to 221.8 mm, 64.6–199.9 mm, 94.8–253.2 mm and 115.5–248.8 mm at year 2005, 2010, 2015 and 2020 respectively. Notably, the CWR_blue_ during the maize growing season was relatively lower in 2010 when compared to the other three study years, attributing to the higher precipitation compared with the other three study years. Additionally, among all the research stations, the CWR_blue_ for 2015 exhibited greater variability compared to the other two years (Fig. 2b).

### 3.2 Water footprints of maize production in the BTH region

**Fig. 3:**
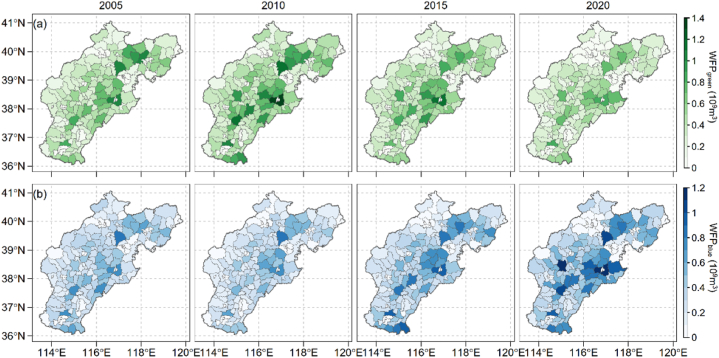
Spatial and temporal dynamics of green water footprint (WFP_green_, a) and blue water footprint (WFP_blue_, b) of maize production in the Beijing-Tianjin-Hebei regions over year 2005, 2010, 2015 and 2020.

Our county-scale WFPs reveal a significant variability within maize-cultivating counties across the BTH region (Fig. 3). Specifically, WFPs for different counties in the BTH region ranged from 6.68 × 10^6^ m^3^ to 1.87 × 10^9^ m^3^, 5.98 × 10^6^ m^3^ to 2.16 × 10^9^ m^3^, and 7.20 × 10^5^ m^3^ to 2.05 × 10^9^ m^3^ and 6.74 × 10^5^ m^3^ to 1.91 × 10^9^ m^3^, for the year 2005, 2010, 2015 and 2020, respectively. The highest WFPs were observed in Tianjin City's Wuqing District for both 2005 and 2010, and in Cangxian, Cangzhou City for 2015 and 2020. Conversely, the lowest WFPs were recorded in Fuxing District of Handan, and Chaoyang District of Beijing for the years 2005 and 2015, and Qiaoxi District of Shijiazhuang for year 2010 and 2020, respectively. WFP_green_ from different counties across the BTH region range from 3.61 × 10^6^ m^3^ to 1.06 × 10^9^ m^3^, 3.74 × 10^6^ m^3^ to 1.39 × 10^9^ m^3^, 3.94 × 10^5^ m^3^ to 1.11 × 10^9^ m^3^, and 2.91 × 10^5^ m^3^ to 0.81 × 10^8^ m^3^ at year 2005, 2010, 2015 and 2020, respectively (Fig. 3a). The regions with the highest and lowest WFP_green_ align with those where the overall WFPs were also the highest and lowest, respectively. Meanwhile, WFP_blue_ which is lower than WFP_green_, ranges from 2.39 × 10^6^ m^3^ to 8.08 × 10^8^ m^3^, 2.24 × 10^6^ m^3^ to 8.12 × 10^9^ m^3^, 3.21 × 10^5^ m^3^ to 9.45 × 10^8^ m^3^, and 3.82 × 10^5^ m^3^ to 1.18 × 10^9^ m^3^ for the year 2005, 2010, 2015 and 2020, respectively (Fig. 3b). Remarkably, the regions with the highest and lowest WF_blue_ values correspond to those with the highest and lowest WFPs, with the exception of 2005 when Lubei District of Tangshan had the lowest WF_green._

### 3.3 Water footprint response to harvest area

**Fig. 4:**
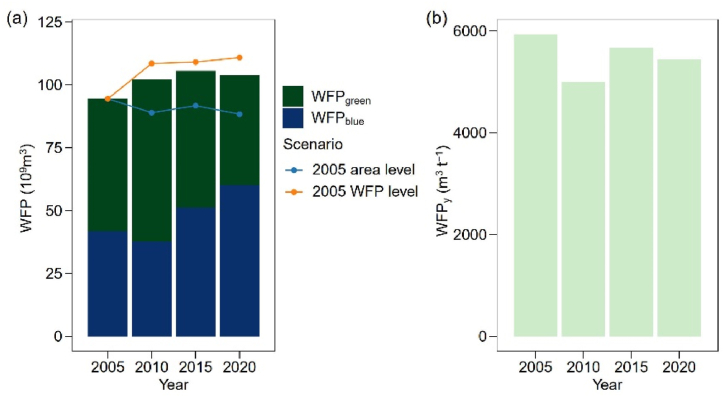
Temporal dynamics of water footprint (WFPs, a) and per maize yield water footprint (WFP_y_, b) of maize production in the Beijing-Tianjin-Hebei regions over year 2005, 2010, and 2015.

The total WFPs for the BTH region were highest in 2015, totaling 1.06 × 10^11^ m^3^, followed by 2020 with 1.04 × 10^11^ m^3^, 2010 with 1.02 × 10^11^ m^3^, and 2005 with 9.44 × 10^10^ m^3^ (Fig. 4a). And the WFP_green_ contribute more than half (55.8 %, 63.2 %, 51.7 % and 42.1 % for year 2005, 2010, 2015 and 2020 respectively) of the WFPs, with only to a small extent, contributed by WFP_blue_ (Fig. 4a). In terms of water footprint per maize production (WFP_y_), we found that the WFP_y_ was highest at year 2005, with 5930.3 m^3^ t^−1^ over the BTH region followed by year 2015 (5670.9 m^3^ t^−1^), year 2020 (5434.1 m^3^ t^−1^) and year 2010 (5001.4 m^3^ t^−1^) (Fig. 4b). To quantify the effect of maize harvest area on WFPs of maize production, we set up a scenario, referred to as the ‘2005 area level’. For this, the harvest area was maintained at the same level as in the year 2005. Under this scenario, we observed that if the maize harvest area for the years 2010, 2015 and 2020 remained the same as that of 2005, the WFPs would decrease by 5.8 %, 3.1 % and 6.6 %, respectively, compared to the current WFPs for the respective years (Fig. 4a).

### 3.4 Water footprint response to climate change

In order to quantify the effect of climate change on WFPs of maize production, we set up a scenario referred to as the ‘2005 WFP level’ scenario. Under this scenario, the crop water requirement for each county was kept the same level in 2010, 2015 and 2020 as in 2005. The WFPs of the BTH region would be 14.9 %, 13.5 % and 15.1 % higher than the current WFPs at year 2010, 2015 and 2020, respectively (Fig. 4a).

### 3.5 Spatial and temporal pattern characteristics of the water footprint

**Fig. 5:**
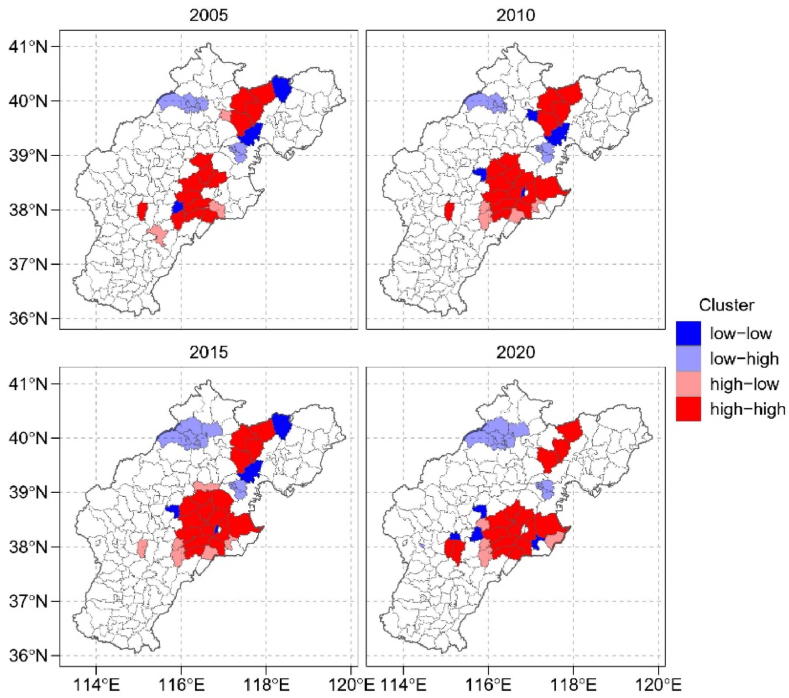
The LISA cluster maps of water footprint for maize production (WFP) of the Beijing-Tianjin-Hebei region over year 2005, 2010, 2015 and 2020.

The specific spatial characteristics of local areas reveal the presence of 14 high-high clustered counties, primarily concentrated in the regions of Cangzhou, Hengshui, Tangshan, Shijiazhuang, and Tianjin City (Fig. 5). Notably, this cluster expanded both westward and eastward during the period from 2005 to 2020, with an increasing number of counties falling into the high-high cluster category. In contrast, the low-low clustered areas appear to be dispersed across the BTH region. In 2005, only three counties exhibited characteristics of low-low clustering, and increased to four counties in 2010 2015 and 2020.

## 4 Discussion

The spatial-temporal analysis of crop water requirement and WFPs aids the identification of priority areas for regulation and the development of appropriate water management strategies. In our study, we found that the green water requirement, although devise within the study period, is higher than the blue water requirement (Fig. 2; Fig. 4). This makes maize production in the BTH region less cost of groundwater compared with winter wheat, which is another main crop in the BTH region with blue water as the dominant source of crop water requirement [37]. During year 2005–2015, the WFP_green_ decreased 9.1 × 10^9^ m^3^ and WFP_blue_ increased 18.4 × 10^9^ m^3^, respectively. And the WFPs over year 2005 to year 2020 increased 9.28 × 10^9^ m^3^. The increased WFPs could be explained by the expansion of the harvest area, which saw an additional 0.5 million hectares in 2020 compared to 2005 (Fig. 1b). This finding was in align with former study which also showed that the total crop water consumption increased with an enlarged production area [14,38]. If the WFPs intensity for the other study years keeps the same as year 2005, the WFPs would rise by at least 10 %, underpinning that the WFPs would increase by the harvest area.

In our study, we found a decline trend for the per yield WFP_y_. The WFP_y_ is influenced by both crop yield and crop evapotranspiration. Previous studies have highlighted that improving crop yield can lead to a reduction in per-yield WFPs and total water consumption [9,14]. Moreover, climate change may regulate plant evapotranspiration by changing the air temperature, rainfall or wind speed, and further influence the WFPs [[39], [40], [41], [42], [43]]. The harvest yield in 2020 showed a 19.7 % increase compared to 2005. This may jointly affect by the improvement of field management, the development of cultivars and the expansion in maize harvest area. Therefore, a decline trend was found for the per yield WFPs. Furthermore, to assess the influence of climate change on the WFPs of maize production, we established scenarios in which the harvest areas for 2010, 2015 and 2020 were held constant with the 2005 level. Under these scenarios, we found that the WFPs decreased for both years. This suggests that climate change, occurring between 2005 and the other three selected research years, had a positive impact on reducing the WFPs of maize production in the BTH region.

The above mentioned increase in WFPs, particularly in blue water suggests that more water resources, especially blue water (irrigation water consumption), are needed for maize production in the BTH region. Our results indicate that reducing the maize cropping area would be a useful way to decrease WFPs. Additionally, increasing the maize yield could improve the water use efficiency of maize production in the BTH region. However, due to the growing population and the need of arable land for food and feed production, it is therefore impossible to diminish maize cropping area in the BTH region. Hence, prioritizing actions such as relocating the maize production areas from regions with high WFPs and WFP_blue_, such as Wuqing District in Tianjin and Cangxian in Cangzhou, or focusing on strategies to increase maize yield, should be considered as key measures to effectively reduce the WFPs. Nonetheless, further investigation into the detailed relocation of maize production and its social and economic effects is still necessary, though it falls outside the scope of this study. It has been reported that future crop-yield gains will increasingly rely on improved agronomic practices and irrigation [44,45]. Considering the water scarcity problem especially the groundwater over-exploitation in the BTH region [46,47], and recognizing the high economic costs associated with irrigation, it becomes imperative to maximize the utilization of green water resources, primarily rainfall, for maize production. To achieve this goal, priority should be given to implementing management practices such as optimizing planting dates, employing straw mulching techniques, and adopting efficient tillage practices. These strategies are essential for enhancing precipitation use efficiency, improving overall green water use efficiency, and ensuring food security in the BTH region [[48], [49], [50]].

## 5 Conclusions

Spatiotemporal analysis of WPFs for maize in the BTH region provide valuable insight into the water usage patterns and further contributing to more sustainable water management practice. In our study, we found that the WFPs in the BTH region range from 5.98 × 10^6^ m^3^ to 2.16 × 10^9^ m^3^. And it showed a rising trend over year 2005, 2010, 2015 and 2020, especially in the eastern central area, where the WFPs exhibited a notably high upward trend. Overall, WFP_green_ attributes mostly to the WFPs of maize production, and WFP_blue_ only contribute to a smaller extent. Additionally, our analysis revealed that the increase of the WFPs of maize over 2005, 2010, 2015, and 2020 was contributed by the extension of the maize harvest area; and the change of plant evapotranspiration, which was induced by changing the air temperature, rainfall or wind speed had a positive effect on reducing the WFPs over the studying period. Our findings highlight the distinct influences of annual fluctuations in climate attributes and variations in harvest areas at the county level. This underscores the need for urgent interventions in specific regions to enhance the sustainability of water usage for agricultural purposes. Moreover, to save the water resource of maize production in the BTH regions, management which could increasing precipitation use efficiency should be considered preferentially e.g., rational planting date and straw mulching.

## Data availability statement

Data will be made available on reasonable request.

## CRediT authorship contribution statement

**Heju Huai:** Writing – original draft. **Qian Zhang:** Methodology, Investigation. **Min Liu:** Data curation. **Xiumei Tang:** Writing – review & editing, Supervision.

## Declaration of competing interest

The authors declare that they have no known competing financial interests or personal relationships that could have appeared to influence the work reported in this paper.
